# The impact of policy perception on technology transfer from boundary-spanning perspective-empirical evidence from Chinese technological enterprises

**DOI:** 10.3389/fpsyg.2022.974436

**Published:** 2022-10-10

**Authors:** Kaiyun Zhang, Qingjin Wang, Xueling Wang, Fengying Zhang

**Affiliations:** ^1^Business School, Qingdao University, Qingdao, China; ^2^Music School, Anshan Normal University, Anshan, China

**Keywords:** policy perception, boundary-spanning, technological potential gap, inter-organizational trust, technology transfer, social capital theory

## Abstract

Technology transfer is an essential source of technological innovation for enterprises, which is conducive to the market transformation of patent achievements and the commercial application of new technologies. Building upon social capital theory, all data analyses were performed using SPSS 22.0 and Amos software with the multiple linear regression method. The study explores the mechanism of policy perception to obtain the technical resources needed for enterprise development through boundary-spanning behavior, with a moderating effect of inter-organizational trust and technological potential gap. The study uses survey data from 125 enterprise teams of 42 technology-based enterprises in China. The results show that policy perceived usefulness and usability significantly promote technology transfer performance and boundary-spanning behavior plays a mediating role between them. Speaking of the influencing factors of technology transfer, technological potential gap significantly moderates the relationship between boundary-spanning and technology transfer performance. In contrast, inter-organizational trust positively moderates the relationship between boundary-spanning and technology transfer performance. The research provides theoretical reference and guidance for enterprises on using policy perception better to improve technology transfer performance in the institutional environment. It also helps inspire enterprises to better deal with the cooperative relationship between relevant stakeholders and achieve win-win cooperation.

## Introduction

Technology transfer is a multilateral behavior intended to improve enterprises’ resource utilization efficiency and opportunities for technological innovation (Grimaldi and Grandi, 2005). It emphasizes the acquisition of technology from sources external to the enterprise and integrating it into current technology (Villani et al., 2017). Sharing technology allows companies to develop new ones, increase the potential value between innovative entities, and create more market opportunities and commercial value for themselves—an important strategy for enhancing technical capabilities and establishing a competitive advantage (Han et al., 2017). Technology transfer is used not only by start-ups, innovative companies, and tech industry giants, but also by existing companies seeking to innovate (Jafari-Sadeghi et al., 2021). The complex process involves exploring, developing, and adopting technology, which is a significant challenge faced by almost all technology-based companies in the current market environment. That is why we believe that it is important both to theory and application to explore the factors that affect how well a company is able to transfer technology.

Existing studies have yet not agreed on a definition of technology transfer. It initially referred to the technology inputs and outputs between different enterprises (Jafari-Sadeghi et al., 2021). As scholars continued to explore and publish, they came to believe that technology transfer includes various concepts, such as the knowledge redistribution, technical knowledge application, geographical field transfer. In this regard, the flow of knowledge resources is essential for technology transfer to be successful (Sahal, 1981); therefore, obtaining valuable unique heterogeneous resources is particularly important. Baker (1990) first introduced the notion of social capital theory to a company’s performance, opening up a line of research on the importance of expanding social networks (Nahapiet and Ghoshal, 1998). Social capital theory believes that capital is embedded in the social networks to which a group belongs and that social relations are an important way to obtain resources (Bolino et al., 2002). Therefore, in exploring the factors that influence the success of technology transfer, scholars looked at the ability and behavior of enterprises to obtain resources through social networks.

Currently, most enterprises in developing countries face issues in the technology transfer process, such as low transfer efficiency, complicated transfer process, high conversion cost, and low yield. Existing research revealed that the root cause of solving these dilemmas lies in improving knowledge and innovation ability, which is an essential path to enhance the competitive advantage of enterprises and deal with technological competition. However, for most technology-based enterprises that lack resources and capabilities, although knowledge gaining and innovation are important, the ability to receive external help is even more precious (Chen et al., 2014). The guidance, support and incentives from government policies are often regarded as important driving forces which can promote the development and growth of those enterprises in their own fields (Li, 2012; Siegel et al., 2007). Only by constantly perceiving the intention of government policies, understanding the connotation of policies, and actively participating in the application of policies, enterprises can benefit from policies and generate a willingness to promote their participation in technology transfer, thereby realizing policy expectations.

Therefore, it is necessary to study the impact of policy perception on technology transfer performance. First, based on the context of most developing countries, policy plays a vital role on those local business development, so the policy factor should be an entry point for further theoretical research. In the process of enterprise technology transfer, government policies are inseparable from enterprise technology innovation activities, and how to improve the positive effect of policies has always been a research hotspot (Bozeman, 2000; O’Shea et al., 2008; Peng, 2013). Second, some scholars used 52 empirical methods, such as structural equation modeling and multiple regression analysis to explore 53 technology transfer performance factors from the perspective of relationships (Wang and Liu, 2022; Yang Z. et al., 2022), including technological innovation networks (Yang and Yang, 2022), organizational proximity (Villani et al., 2017), and subject interaction (Scuotto et al., 2020). In terms of knowledge, such factors include knowledge networks (Marrocu et al., 2013) and knowledge collaboration (Villani et al., 2017), and in terms of structure, they include endogenous evolutionary mechanisms and spatial correlation characteristics of networks (Broström, 2010; Slavtchev, 2013), while rarely explored it from the perspective of policy perception.

The research on the involvement of policy perception has practical significance. In reality, many technology transfer enterprises are limited by their own resources and capabilities and are unable to achieve technological transformation through technology transfer in the short term. However, based on the trend of sustainable development, the enterprises themselves are concerned about long-term development and eager to get the help of external forces, especially the support from the government (Siegel et al., 2007). Therefore, exploring the impact of corporate policy perception ability on technology transfer performance will promote policy-making pertinence and effectiveness.

A considerable number of studies have highlighted boundary-spanning that explain the relationships between ability and performance (Zhang X. et al., 2019; Liu et al., 2016). However, there has been little research that examine the technology transfer processes that boundary-spanning can facilitate. Social capital theory states that organizations continually exchange and share resources through relationship networks to enhance their potential value (Adler and Kwon, 2002). Based on this theory, our study contend that boundary-spanning behavior is a mediator effect for enterprises that helps them continually build their network of relationships, enhance relationship capital and each other’s willingness to transfer technology, and thus improve technology transfer performance. The technological innovation enterprises in the institutional environment still have the disadvantage of resource acquisition, and the research proves that the keen policy perception stimulates enterprises to continuously obtain high-quality external resources to alleviate resource constraints in order to achieve the strategic goal of technological innovation (Peng, 2013). Boundary-spanning behavior is helpful to solve this dilemma. Consequently, exploring the impact of policy perception on boundary-spanning is crucial to create a holistic understanding of how policy perception influences technology transfer performance. In addition, the value of boundary-spanning for an enterprise’ innovative performance has been well documented (e.g., West, 2000; Tjosvold et al., 2004; Schippers et al., 2015). We wonder whether boundary-spanning behavior have the same stimulatory effect on firm technology transfer performance. Therefore, we seek to explore the mediating role of boundary-spanning between policy perception and technology transfer performance.

Previous studies have explored the influencing factors that moderate the relationship between boundary-spanning behavior and performance, such as environmental uncertainty and goal-oriented strategy (Yao, 2019; Zhang X. et al., 2019). Most of the previous researches focus on the external environment of enterprises, while few or no researches focus on the influence of the boundary crossing and the performance of technology transfer from the internal subject level of enterprises, such as the technology potential difference and trust. Technology transfer performance is inseparable from the interaction between the entities involved. Social capital theory reveals that “heterogeneous groups” tend to be excluded from social networks and that people gravitate toward “homogeneous groups” that are not very different from themselves (Portes and Sensenbrenner, 1993; de Vaan et al., 2019). In other words, while transferring technology, companies are more inclined to choose companies that are not much different from their own technology potential (Mattes, 2012; Steinmo and Rasmussen, 2016). That is to say, enterprises with different technological potentials often result in an unequal exchange of technological resources, which may cost more to maintain and have a negative effect on technology transfer performance. Furthermore, trust is another critical dimension of social capital theory. Trust is a fundamental element of cooperation, an important mediator variable in the research that cannot be ignored. Following prior research, we believe inter-organizational trust exists when one party has confidence in the honesty, reliability, and integrity of their partners (Seppänen et al., 2007). Inter-organizational trust plays a major role in embedding the variable into the technology transfer performance model constructed by research.

Overall, building on social capital theory, our study attempted to explore how enterprise policy perception impacts technology transfer performance from the role of policy perception combined with boundary-spanning, an important enterprise behavior variable, by focusing on technology-intensive enterprises. We further investigate the moderate effect of potential technological gap and trust in the process. The study further enriches the theoretical contextual research of social capital theory to provide theoretical support for promoting the practice of enterprise technology transfer.

## Theoretical background and hypothesis

### Policy perception and technology transfer performance

With the in-depth study of technology transfer by scholars, evaluations of technology transfer performance indicators have gradually changed from focusing only on income (Zeng et al., 2020) (e.g., actual income of enterprises after technology transfer) to subsequent technology transfer potential (e.g., number of technology transfer personnel, scientific research funding, number of papers or patents published (Zhang Y. O. et al., 2019) to, eventually, the entire technology transfer process (e.g., accumulation of technical resources, technological discoveries (Yang Z. et al., 2022). The evaluation of technology transfer performance in existing research has gradually become multidimensional and complex. Technology transfer inputs and outputs undoubtedly cause complex, dynamic institutional problems for enterprises (Bruneel et al., 2010); hence, enterprises must improve their perception of the institutional environment, including the institutional itself, institutional change, and institutional response. Such perception helps them make decisions conducive to business development based on the characteristics of the enterprise’s own development and the content of the system. Policy perception therefore plays an important role in the process. In order to promote technology transfer among enterprises, the government has formulated coherent technology transfer incentives. Enterprises perceive policies in terms of resource allocation, awards, and financial returns (Ni and Zhang, 2021).

In terms of resource allocation, enterprises with strong policy perceptions are able to perceive the government’s efforts as promoting a certain behavior with greater financial and social resources and thus are able to respond more actively to policy calls and bring their development strategy in line with government resource allocation (Chiang, 1991). In this case, it is beneficial for enterprises to alleviate resource constraints associated with technology transfer. In terms of evaluation allocation, enterprises with strong policy perceptions adopt a positive attitude to technology when governments honor outstanding contributors to technology transfer (Fu, 2019). This positive perception motivates companies to put efforts into technology transfer, which promotion indirectly improves their professionalism (Judge and Zapata, 2015). At the same time, innovative behavior is conducive to winning the trust of enterprises in the same industry, thus expanding their corporate social network and resource acquisition channels and helping to enhance their social capabilities. Increasing social capital improves the potential of technology transfer and reduces potential technology transfer paths. Financial return is the government’s “compensation” to companies with outstanding technology transfer performance (Nordhall and Knez, 2018). Enterprises with strong policy perception gain access to potential policy advantages, seizing the opportunity for development and winning the benefits of the policy. Financial returns also help enterprises introduce outstanding candidates to their company and better apply technology to research and development (Ni and Zhang, 2021), which in turn improves technology transfer performance. In this way, policy perception plays a critical role in helping enterprises gain valuable insight from the government, including financial returns, knowledge, protection, and opportunities. Enterprises with strong policy perception are thus able to discern more opportunities, enrich technology transfer performance by easing resource constraints, improve technology research and development capabilities, and reduce the complexity of technology transfer. Hence, we propose the following hypothesis,

*H1*: Policy perception positively affects enterprises’ technology transfer performance.

### The mediating role of boundary-spanning behavior

In this study, boundary-spanning behavior refers to the interactive actions between the enterprise technology transfer team and external members of the enterprises (Xu et al., 2022). After the government promulgates the technology transfer incentive policy, enterprises perceive usefulness based on policy objectives, content, and enjoyment conditions according to their own characteristics. Moreover, enterprises form a usability perception based on technical resource supply and cost measurement in the process of policy implementation. As early Davis (1989) proposed the concept of the technology acceptance model, which pointed out that willingness stems from “behavioral intentions,” and attitudes determine personal behaviors. That is to say, perceived usefulness and perceived usability evoke behavioral attitudes which can be later transformed into behavioral intentions. And the establishment of goal drives organizations to produce out-of-boundary behaviors and look for ways to obtain the resources needed to achieve goals (H and Zhu, 2020).

In addition, some studies have pointed out that policy perception plays an important role in the transformation of target intentions into real behavior, which could be an antecedent variable or an intermediate variable (Su and Geng, 2014). These studies provide a sufficient basis for us to deeply examine the impact mechanism of policy perception and boundary-spanning behavior. Based on this, we believe that when companies have a strong perception of the usefulness and usability of policies, they will be more sensitive to economic benefits (Dacin et al., 2010; Korschun, 2015), and they will expect to obtain more benefits from policy support, which also means that companies need to complete a complex and innovative technology transfer goals. It is difficult to complete the task independently by relying only on the limited resources, knowledge and information of the internal team to a large extent (Gong et al., 2013). By crossing borders, enterprises have interactions with other resources, which is conducive to promoting knowledge flow and sharing, and promoting quality of technology transfer activities. In addition, the boundary-spanning behavior is conducive to the formation of technology alliances between enterprises, providing opportunities for further technology transfer cooperation (Luo and Liu, 2022). It can be seen that, driven by the goal of the enterprise technology transfer, different parties will tend to carry out interaction and collaboration beyond the organizational boundary (Teigland and Wasko, 2003; Grand et al., 2016), and obtain the required resources, thereby promoting the occurrence of boundary-spanning activities (Papanastassiou et al., 2020).

*H2*: Policy perception has a positive effect on the boundary-spanning behavior of enterprises.

Baker (1990) first introduced the notion of social capital theory, opening up a line of research into whether social interaction spurs the exchange of resources. Social networks are an important way to obtain resources, and boundary-spanning provides the preconditions necessary for interactions between enterprises and external parties (Cohen et al., 2002; Van de Ven et al., 2007). In our study, we proposed that technology transfer teams conduct technical exchanges and cooperate with outside firms through boundary-spanning behaviors, which are conducive to the exchange of technology transfer information and storage of technical knowledge, thus increasing social capital. A large number of studies have confirmed that social capital is an important condition for improving enterprise performance (Armstrong et al., 2015).

First, by establishing technology transfer partnerships with other enterprises through boundary-spanning behavior, enterprises can obtain additional resources (Druskat and Wheeler, 2003) and help reduce cost and risk (Rindova and Kotha, 2001). For example, cooperation between enterprises can help them cope with emergencies that arise during technology transfers. They can discuss how to reduce technology transfer costs more effectively. Second, boundary-spanning allows enterprises to collect information, such as the capabilities, specialties, technical requirements, and previous progress of different enterprises (Janssen and Abbasiharofteh, 2022). When faced with highly innovative, complex technology transfers, boundary-spanning can be a way to consult more experienced enterprises. Scholars have pointed out that communication and sharing experiences improves performance significantly (Liu et al., 2021). Finally, market dynamics have a major impact on the success of technology transfers. Boundary-spanning behavior allows enterprises to obtain more policy information about technology transfer in the industry from different enterprises, so as to respond promptly and effectively to potential risks and threats caused by environmental fluctuations, thereby reducing risk.

Therefore, we put forward the following hypothesis,

*H3*: Boundary-spanning behavior has a positive effect on technology transfer performance.

Combining the analysis of Hypothesis 2 and Hypothesis 3, we believe that when the company has a strong policy perception ability, it can quickly obtain public and policy information (e.g., invesntment policy, taxation policy) related to the technological innovation transfer of enterprises (Yang Z. et al., 2022), and they have a better interpretation of the policy objectives, purpose, content, conditions of use, the ease of measurement and cost of resources. Social capital theory reveal that there is a collective understanding (e.g., collective values, collective norms, and collective culture) shared by different subjects in the external institutional field when economic actors carry out corresponding economic behaviors (i.e., setting strategic goals and implementing tactical behaviors) in their organizational structure positions (Granovetter, 2018; Uzzi, 2018). In this regard, when enterprises have a strong perception and understanding of policies, they can better perceive government policy intentions, understand policy connotations, and actively participate in policy application. According to the Technology Acceptance Model (Davis, 1989), a strong perception also motivates enterprises to carry out boundary-spanning behaviors to achieve the strategic goals of technology transfer and to cope with the complex challenges of technological innovation. The boundary-spanning behavior can enhance mutual resource sharing willingness and behavior by initiating horizontal interactive activities to the relevant technology transfer subjects, which is conducive to obtaining the complementary resources required for the activity (Luo and Liu, 2022). Obtaining more policy and development information about technology transfer helps enterprises effectively deal with the potential risks and threats brought by environmental volatility to enterprise technology transfer, reduce the complexity of technology transfer, and improve the performance of technology transfer. Based on this, we propose the following hypothesis,

*H4*: Boundary-spanning plays a mediating role between policy perception and technology transfer performance.

### The moderating effect of technological potential gap

The technological potential gap refers to the gap in knowledge and technology in a technology transfer (Cui et al., 2014), including gaps in knowledge reserves, product quality, structure, and knowledge domination (Haddad and Harrison, 1993). The gap highlights the importance of the flow of technical resources between enterprises. One may wonder to what extent the technological potential gap affects technology transfers between enterprises. We speculated that when one enterprise is of high status, it often possesses leading knowledge resources for technological innovation, while other enterprises at the same level as each other can form a technological alliance with higher-status enterprises through boundary-spanning behaviors, creating a strong, powerful whole and promoting enterprises within the industry. Such actions also help improve technology transfer performance (Wang et al., 2022). On the other hand, companies with lower technological potential tend to “imitate and follow,” a strategy with a low degree of innovation that makes it difficult to establish business contacts with higher-potential companies (Heller, 1985). They therefore tend to cooperate with companies at their own level, and they progress and grow together. In the third scenario, lower-status companies are more willing to work with higher-status companies in order to learn advanced technical concepts (Li and Liu, 2012; Fahad et al., 2022). However, when there is a large technological potential gap, low-potential enterprises may need to pay high relationship maintenance costs (Hansen, 2002), a huge economic burden. Hence, the gap may be detrimental technology transfer performance. Scholars (Jaffe, 1986; Griliches, 1991) found that when enterprises have relatively low technological potential, their knowledge and technical foundation are average within the industry, leaving them plenty of room for technological improvement. Through boundary-spanning activities, enterprises offer each other valuable, diverse technical resources, helping them achieve breakthroughs and innovation at their original level (Porter and Heppelmann, 2015; Chen et al., 2020; Zhou and Cheng, 2021), thus improving the enterprise’s technology transfer performance. In addition, social capital theory reveals that “heterogeneous groups” tend to be excluded from social networks and that people gravitate toward “homogeneous groups” that are not very different from themselves (Zhan et al., 2022). This phenomenon shows that, in the process of technology transfers, companies are more inclined to cooperate with those with similar technological potential (Liu and Shan, 2013).

Based on the above analysis, we found that when there is a large technological potential gap between enterprises, boundary-spanning between enterprises reduces the degree of technology transfer between enterprises accordingly and affects the effective replication and reconstruction of technical knowledge, such as resources and other intellectual capital, by enterprises. On the other hand, a large technological potential gap increases the cost of maintaining the relationship between enterprises (Ren et al., 2015), which is not conducive to improving technology transfer performance. In this way, when the technological potential gap between enterprises is small, technical capabilities are better matched. For enterprises with higher potential, boundary-spanning is conducive to the formation of strong alliances and professionalization in the industry to win prestige; for those with lower potential, boundary-spanning can help enterprises absorb new knowledge and technology, make it easier to break through technology transfer bottleneck, and achieve better technology transfer performance. As a result, our paper proposes the following assumptions.

*H5*: The technological potential gap negatively moderates the relationship between boundary-spanning and technology transfer performance.

### The moderating effect of trust

Due to its abstraction and complexity, scholars have not reached a consensus on the definition of trust (Hosmer, 1995; Bidault, 1997; Rousseau et al., 1998). In psychology, trust refers to “a psychological state comprising the intention to accept vulnerability based upon positive expectations of the intentions or behavior of another” (Nordhall and Knez, 2018). In short, trust refers to the degree to which members expect each other to be relied upon according to their own inner predictions (Zaheer et al., 1998). Consistent with this logic, many empirical studies have demonstrated that trust is the basis of transactions or exchanges between team members and plays an important role in maintaining the stability of team cooperation (Creed et al., 1996; Blomqvist, 2002). Companies build partnerships based on trust, making it easier to build a sense of responsibility in teamwork (Plank et al., 1999). There are also scholars who have found that relational trust and calculative trust both have a positive effect on cooperative performance (Miles et al., 2000). Social capital theory states that trust, as a normative social capital, is conducive to the establishment of similar values or strengthening of existing values among members, and of normative conventions, which are conducive to the establishment of friendly and mutual assistance networks and reduce the risk of cooperation (Ring, 1996; Zaheer and Harris, 2005; Bromiley and Harris, 2006).

Trust is a work atmosphere shared by members of an organization. We speculate that when enterprise technology transfer teams at a higher level of trust, they are easier to feel the sincerity among collaborators, which will bring psychological security to organizational members and promote innovative behavior via boundary-spanning activities. Trust climate encourages innovators to freely express innovative ideas, which in turn, is conducive to the improvement of technology transfer performance (Verburg et al., 2018). Second, higher organizational trust makes teams have a higher willingness to abide by the technology transfer agreement, such as the agreed responsibilities of all parties and how to carry out the technology transfer cooperation content in detail, which further enhances the willingness of organizations to further cooperation as well as establish long-term technical cooperation alliance relationship (Granovetter, 1985; Ring and Van de Ven, 1994; Gulati, 1995). As a result, it is beneficial to reduce the relationship maintenance cost and promote the performance of technology transfer (Bidault and Jarillo, 1997). In addition, studies have shown that a higher organizational trust environment is conducive to the formation of members’ organizational identity and team cohesion, and it promotes team members to contribute to the achievement of enterprise technology transfer goals, and even willing to take the initiative to undertake challenging work Afsar and Masood (2018). Therefore, we hypothesize that in a high-trust environment, technology transfer performance will been increase through innovative activities between parties via boundary-spanning behavior. On the contrary, when enterprises in a low-level trust environment, it is easy to generate opportunism or doubt the content of cooperation in cross-border activities of enterprises, which may terminate cooperative relationship, which is not conducive to technology transfer performance (Aryee, Budhwar and Chen, 2002).

*H6*: Trust positively moderates the relationship between boundary-spanning and technology transfer performance.

**Figure 1:**
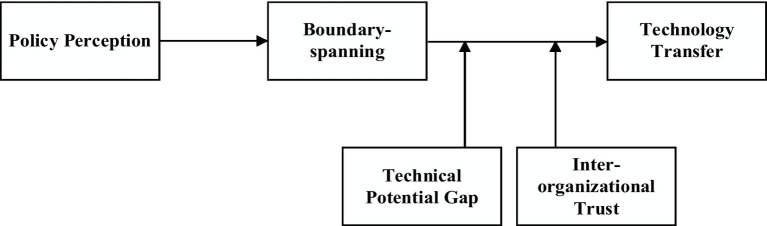
Theoretical analytical framework.

Based on these assumptions, this paper has constructed the theoretical model shown in Figure 1.

## Materials and methods

### Calculate sample size

This content was not included in the first draft due to lack of space. We performed power analysis using G*Power 3.1 software to determine the appropriate sample size required for this study (Faul et al., 2009). Specifically, we estimated the sample size needed based on a regression model with nine explanatory variables (five control variables, one independent variable and one mediator variable plus two moderator variables, totaling nine explanatory variables) assuming an effect size of f2 = 0.15, an alpha error of 0.05, and beta power of 0.95. The resulting minimum sample size was calculated to be 166 and actual power reached more than 95 percent. This suggests that our study required a minimum of 166 samples to detect effects. Finally, we collected 402 questionnaires, which met the minimum sample requirements.

### Sample and data sources

Our survey respondents take 125 technology transfer teams from 42 representative technology-intensive enterprises in China, including seven provinces, Shaanxi, Jiangsu, Fujian, Shandong, Guangdong, Shanghai, and Anhui. The industries include microelectronics, electronic information technology, space science, aerospace technology, energy and new energy technologies. The main reason why these companies were selected were their characteristics that they have strong technological and economic strength, have experience in technical exchanges, have technical cooperation with domestic-related companies or foreign technology companies, and have received the country’s policy support. The respondents are representative of the research.

The research data were collected for 22 months, from January 2019 to October 2020. Our research team conducted a survey on multiple technology transfer companies first, then we explained the research purpose clearly to the respondents and promised the confidentiality of the research. Under the managers’ guidance, we had tours of the companies to understand the company’s basic situation, which laid the foundation for future research and cooperation. We mainly use face-to-face interviews, supplemented by emails. To avoid possible risks, we conducted pre-research piloted in a randomly selected company and then adjusted the questionnaire’s content according to the bias that emerged.

The questionnaire collection was divided into two stages. Based on our experience, questionnaires should be collected at least three months apart to reduce homology bias and measure causality more precisely (Podsakoff et al., 2003). In the first stage, respondents fill in demographic information and the questionnaires for independent, mediating, and moderating variables. Then, we bound, classified and coded the questionnaires of the same company to facilitate the development of the second questionnaire. Our second questionnaire collection started six months later. The content of the dependent variable of the questionnaire was distributed to the corresponding personnel according to the previous coding situation and enterprise demographic information. Finally, we matched the questionnaire data collected in the two stages, eliminated apparent errors, omissions and invalid questionnaires that were answered randomly, and finally collected 402 valid questionnaires. The effective rate of the questionnaire reached 80.4%.

The scale of enterprises ranges from 50 to 200 people, and the proportion with 100 to 150 employees is the largest among our sample, accounting for 39.1% (SD=1.1). The proportion of men is much higher than that of women. The ratio is close to 8:1. All respondents worked in their current company for 2.63 years ([SD]=1.049).

### Measures

Except for general personal information from the respondents, the study rated all questionnaire indicators using a Seven-point Likert scale from 1, “completely disagree,” to 7, “completely agree.” We use mature questionnaires developed by other scholars to test our variables and translate all the questions from English to Chinese (Brislin, 1986). In order to better adapt to the research situation of Chinese enterprises, we have made appropriate modifications to the questionnaire under China’s context.

#### Dependent variable

Technology transfer performance was measured using a six-item scale that reflected the two proposed performance dimensions: vertical technology transfer performance and horizontal technology transfer performance (Yli‐Renko, Autio and Sapienza, 2001). The content of the items includes, “The conversion rate of technological achievements of enterprises has been greatly improved”, “The technical and economic benefits of enterprises have been greatly improved”, and “The technological innovation capabilities of enterprises have been greatly improved.” The alpha reliability (α) for this scale was 0.90.

#### Independent variables

We draw on the policy perception scale developed by past scholars (Acedo and Jones, 2007; Peng et al., 2013) and adjust the questionnaire’s content according to the situation of Chinese enterprises. The content of the scale has five items, including “Can make a good trade-off between the cost and benefit of implementing the policy content?” and “Can perceive the policy well to promote the technical exchange and sharing among different subjects?” And so on. The content of the scale can well measure the perceived ability of enterprises to policy (α=0.85).

#### Mediator variables

Boundary-spanning Scale in the study used to measure the cooperative behavior between companies. We combine mature scales used in three studies *via* Ancona and Caldwell (1992), Brion et al. (2012), and Xu et al. (2022). The scale contains four items, including “Interacting and collaboration with outside of organization enable employees to complete specific project works with a high quality,” and “Communication and collaboration with the outside of the organization can provide practical suggestions for our project implementation.” A result of α = 0.90 indicates the scale has high consistency and stability.

#### Moderator variables

We use the scale developed by Stucki and Woerter (2017), and Dai and Lin (2018) to test the potential technical gap. The questionnaire includes five research contents such as “There are differences between other companies and us in the field of professional technology” and “There are differences between other companies and us in the level of scientific research and technology.” A result of α = 0.95 reflects a high internal consistency.

Referring to a mature scale developed by scholars McAllister (1995), the scale was modified based on Chinese enterprises’ situation, and the consistency of the scale has been tested. The final scale included eight items such as “We can share ideas without boundary” and “We share questions with members in our cooperative organization, and I believe they will provide valuable answers,” with a score of α = 0.97.

#### Control variables

We controlled multiple factors impacting the enterprise’s technology transfer performance. We controlled for the demographic characteristics of the respondents and industry characteristics (e.g., gender, years of employment (Zhou, 2022), firm size (i.e., as it affects technological development (Wu and Lin, 2022), firm establishment time (i.e., as it affects technological maturity (Zhou et al., 2020), and the level of economic development in the region where the company is located (i.e., as it affects the technical level of the enterprise) (Duan et al., 2018; Chen et al., 2022).

#### Model design

Combining theoretical deduction and research hypothesis, our paper establishes the model as follows:M = α1 + α2X + αiControl_gender,years on the job, enterprise scale, enterprise age regional development_ + ε (1).

Y = α’1 + α’2 X + α’3 M + α’4 T1 + α’5 T2 + α’6 M × T1 + α’7 M × T2+ α’iControl _gender, years on the job, enterprise scale, enterprise age regional development_ + ε (2).

The explained variables are technology transfer performance (Y), boundary spanning (M). The main explanatory variables are policy perception ability (X), technology potential gap (T1), inter-organizational trust (T2) and interaction term (M × T1, M × T2).

The coefficient α2 in Equation (1) represents the correlation between policy perception and boundary-spanning; Control stands for Control variable. Similarly, in Equation (2), coefficient α’2 represents the correlation between policy perception and technology transfer performance, coefficient α’3 represents the correlation between boundary-spanning and technology transfer performance, coefficient α’4 represents the correlation between technological potential gap and technology transfer performance. α’5 represents the correlation between inter-organizational trust and technology transfer performance, α’6 represents the impact of boundary-spanning and technology potential gap interaction term on technology transfer performance, α’7 represents the impact of boundary-spanning and inter-organizational trust interaction term on technology transfer performance. This paper will conduct hierarchical regression analysis on models (1) and (2) to verify hypotheses 1 to 4, and moderating effects between hypotheses 5 and 6.

## Results

### Common methods variance test

In order to test the problem of common method bias, the study used Harman’s one-way variance test to conduct an unrotated factor analysis on all items of the questionnaire. The variance explained by the first principal component is 25.22%, which does not account for half of the 74.89% of the total variance explained, indicating that the problem of common method bias has little impact on the results of this study. The results are shown in Table 1.

**Table 1:** Total variance explained.

Factor	Initial eigenvalues			Extraction sums of squared loadings		
	Total	% of variance	Cumulative %	Total	% of variance	Cumulative %
1	7.06	25.22	25.22	7.06	25.22	25.22
2	6.14	21.91	47.13	6.14	21.91	47.13
3	3.75	13.40	60.52	3.75	13.40	60.52
4	2.13	7.61	68.13	2.13	7.61	68.13
5	1.89	6.76	74.89	1.89	6.76	74.89

Extraction method: principal component analysis.

To verify the discriminant validity of each construct of the model in this study, confirmatory factor analysis (CFA) was conducted using Mplus 7.4 software. Assessing Discriminant Validity Prior to hypothesis testing, we first conducted a CFA from an enterprise perspective by using aggregated scores of five key scale constructs: policy perception, boundary-spanning, technological transfer performance, technical potential gap and trust. The results of the CFA suggested that the expected five-factor model fits our data reasonably well [χ2 (402) /Df = 1.7, *p* < 0.001; root-mean-square error of approximation (RMSEA) = 0.04; standardized root mean residual (SRMR) = 0.03; comparative fit index (CFI) = 0.97]. The fit was superior to other models. Taken together, these results favored the five-factor model, thus supporting discriminant validity among the measures. The results are shown in Table 2.

**Table 2:** Confirmatory factor analysis result.

Factorial Modeling	χ^2^	Df	χ^2^/Df	CFI	GFI	TLI	IFI	RFI	RMSEA	SRMR
Five-factor model	576.51	340.00	1.70	0.97	0.91	0.97	0.97	0.93	0.04	0.03
Four-factor model^a^	2468.03	344.00	7.17	0.76	0.65	0.74	0.77	0.71	0.12	0.12
Three-factor model^b^	3502.73	347.00	10.09	0.65	0.56	0.62	0.65	0.59	0.15	0.17
Two-factor model^c^	4333.22	349.00	12.42	0.56	0.48	0.52	0.56	0.50	0.17	0.20
One-factor model^d^	5651.37	350.00	16.15	0.41	0.37	0.36	0.41	0.35	0.19	0.23

*N* = 402. a, combine technology potential and trust into one underlying factor; b, combine technology potential, trust, and boundary-spanning into one potential factor; c, combine policy perception, boundary-spanning, technological potential, and inter-organizational trust into one potential factor; d, attribute all items to the same underlying factor.

### Reliability and validity analysis

A frequent concern in a questionnaire survey is whether the overall reliability and internal consistency reliability meet the standards to reflect the stability of the questionnaire results. Cronbach’s coefficient was used to measure the test in the current research. We use SPSS22.0 to calculate the Cronbach’s coefficient of the overall questionnaire with a result of 0.914. Each variable of internal items was more significant than 0.8, indicating that the scale has good overall reliability and internal consistency reliability.

The factor loadings of the questionnaire data were analyzed by Amos22.0 software. The result showed that the factor loadings were all greater than 0.5, the combined reliability CR was greater than 0.8, and the average variance variation AVE of each latent variable was greater than 0.5, indicating that the model support explained the construct well. The specific results are shown in Table 3.

**Table 3:** Scale items, reliability, and validity results.

Variables	Items	Standardized coefficients	Cronbach’s alpha	AVE	CR
Policy perception	We have a good trade-off between the costs and benefits of policies related to technology transfer	0.72	0.85	0.53	0.85
	We can perceive that the policy content is specific and feasible, and it is very attractive to enterprises	0.70			
	We feel that the policy is helpful in promoting technology sharing and exchange among different innovators	0.72			
	We feel that the policy looks good and can be implemented well	0.70			
	We feel that policies can effectively address practical problems in the process of technology transfer	0.78			
Boundary-spanning	Communication and collaboration with the outside world can enable enterprise members to complete specific project work with high quality	0.85	0.90	0.69	0.90
	Communication and collaboration with the outside world can provide practical advice on project implementation	0.81			
	Communication and collaboration with the outside world can enable enterprise members to successfully deal with the challenges and difficulties involved in other professional fields in the project	0.82			
	Communication and collaboration with the outside world can enable enterprise members to learn knowledge and technology outside the field	0.85			
Technology transfer performance	The conversion rate of technological achievements of enterprises has been greatly improved	0.77	0.90	0.61	0.90
	Technology transfer has brought about a substantial increase in the direct economic benefits of enterprises	0.75			
	After technology transfer, the technological innovation capability of enterprises has been greatly improved	0.77			
	The social benefits brought by enterprises after technology transfer have been greatly improved	0.79			
	The development speed and product quality of enterprise-related products have been greatly improved	0.78			
	After the technology transfer, the market share of the enterprise or the level of new market development has been greatly improved	0.81			
Technological potential gap	There are differences between us and other companies in the field of professional and technical exchanges	0.89	0.95	0.79	0.95
	There are differences between us and other companies in the level of scientific research and technology	0.88			
	There are differences between us and other companies in the technical level of the main business	0.89			
	There are differences between us and other companies in the transformation and application of technological achievements	0.89			
	We differ from other companies in the quantity and quality of technical knowledge	0.91			
Inter-organizational trust	We are free to share ideas	0.90	0.97	0.79	0.97
	There will be a sense of loss if we can no longer work with members of the organization	0.86			
	If we share questions with cooperating organization members, I am sure they will constructively answers	0.89			
	We make substantial emotional investments in our working relationships	0.89			
	We believe that members of the organization approach their work with professionalism and dedication	0.91			
	We have no reason to doubt the organization members’ ability to work and their readiness to work	0.89			
	We trust that co-op members will not make our work more difficult by being careless at work	0.90			
	Our partners have all had business connections with members of this organization and we trust them	0.91			

### Descriptive statistics

The current study takes the manager’s gender, working years, age, scale and regional development level of business leaders as control variables and uses SPSS 22.0 software to conduct descriptive statistical analysis on all variables designed. The means, SDs, and correlations for all the team variables are shown in Table 4. As Table 4 reveals, technology transfer performance is positively correlated with both team policy perception ability (r = 0.424, *p* < 0.01) and boundary-spanning behavior (r = 0.01) 0.378, *p* < 0.01). Boundary-spanning behavior was also significantly correlated with policy perception (r = 0.232, *p* < 0.01).

**Table 4:** Mean, standard deviation, correlation coefficient and AVE value of each main variable.

	1	2	3	4	5	6	7	8	9	10
1. Gender	–									
2. Years on the job	−0.092	–								
3. Enterprise size	−0.174^**^	0.093	–							
4. Enterprise age	−0.040	0.288^**^	0.021	–						
5. Regional development	−0.063	−0.036	−0.008	−0.070	–					
6. Policy perception	−0.063	0.049	0.015	0.086	−0.010	0.728				
7. Boundary-spanning	0.025	0.057	−0.003	0.018	0.072	0.330^**^	0.831			
8. Technology transfer performance	−0.029	0.088	0.071	0.131^**^	−0.076	0.424^**^	0.378^**^	0.781		
9. Technological potential gap	−0.077	0.054	0.012	−0.002	0.045	−0.039	0.048	0.093	0.889	
10. Inter-organizational trust	0.005	0.030	0.011	0.047	0.125^*^	0.028	0.043	−0.077	0.232^**^	0.889
11. Means	0.63	2.63	70.15	2.59	1.64	5.16	5.00	5.20	4.42	4.68
12. SDs	0.482	1.049	1.1	0.9	0.707	0.87	1.15	0.95	1.45	1.44

*N* = 402, ** represents *p* < 0.01, * represents *p* < 0.05.

### Hypotheses testing

Hierarchical regression was adopted to verify the research hypothesis in this paper, and a total of 10 regression models were constructed. The results are shown in Table 5. As indicated in Model 4, after including the controls, policy perception was positively related to technological transfer performance (*b* = 0.45, *p* < 0.001); thus, Hypothesis 1 was supported.

We followed the procedures established by Baron and Kenny (1986) to test Hypothesis 2 regarding the mediating role of boundary-spanning behavior in the policy perception of technological transfer performance. First of all, we verified that policy perception positively correlated with technological transfer performance. Next, policy perception as evaluated by followers was positively related to boundary-spanning behavior (*b* = 0.44, *p* < 0.01; Model 2), Hypothesis 2 is verified. Based on model 5, boundary-spanning behavior positively affects the performance of enterprise technology transfer (*b* = 0.32, *p* < 0.01), thus significant result supported Hypothesis 3. Finally, in Model 6, in which boundary-spanning behavior was added, the effect of policy perception on technological transfer performance became less significant (*b* = 0.35, *p* < 0.01). We conducted a bias-corrected bootstrap analysis (5,000 samples) with the PROCESS macros developed by Hayes and Scharkow (2013) to shed light on the indirect effects further. We found that the indirect effect of policy perception on technological transfer performance *via* boundary-spanning behavior was 0.11, with a 95% CI [0.03, 0.21]. The results revealed that boundary-spanning behavior was a partial mediator, supporting Hypothesis 4.

**Table 5:** Hierarchical regression result.

Explanatory variable	Boundary-spanning		Technology transfer performance
	M1	M2	M3	M4	M5	M6	M7	M8	M9	M10
Control variable										
Gender	0.08	0.13	−0.03	0.02	−0.05	−0.01	−0.04	−0.03	−0.05	−0.05
Years on the job	0.07	0.06	0.04	0.03	0.021	0.02	0.02	0.03	0.02	0.02
Enterprise scale	−0.00	−0.00	0.05	0.05	0.053	0.05^*^	0.05	0.06	0.05	0.05
Enterprise age	0.01	−0.02	0.12^**^	0.08^*^	0.114^**^	0.09^*^	0.12^**^	0.11^*^	0.12^**^	0.11^**^
Regional development	0.13	0.13^*^	−0.09	−0.09	−0.13^**^	−0.11	−0.13^**^	−0.13^*^	−0.11^**^	−0.13^**^
Independent variable										
Policy perception		0.44^***^		0.45^***^		0.35^***^				
Mediating variable										
Boundary-spanning					0.32^***^	0.23^***^	0.31^***^	0.29^**^	0.32^***^	0.35^***^
Moderator variable										
Technological potential gap							0.05^*^	0.06^*^		
Inter-organizational Trust									−0.06^**^	−0.07^**^
Interaction terms										
Boundary-spanning × Technological potential gap									−0.11^**^		
Boundary-spanning × Inter-organizational trust											0.11^***^
*R* ^2^	0.01	0.12	0.03	0.20	0.17	0.27	0.18	0.20	0.18	0.21
*R*^2^ variation	0.01	0.11	0.03	0.17	0.15	0.07	0.01	0.03	0.01	0.03
*F*	0.80	8.95^**^	2.31^**^	16.28^**^	13.78^**^	20.27^**^	12.25^**^	12.56^**^	12.43^**^	13.14^**^
*F* variation	0.80	49.17^**^	2.31^**^	83.77^**^	69.10^**^	35.65^**^	2.74^*^	12.25^**^	3.75^*^	15.03^**^

*N* = 402, ***p < 0.01, **p < 0.05, *p < 0.1.

**Figure 2:**
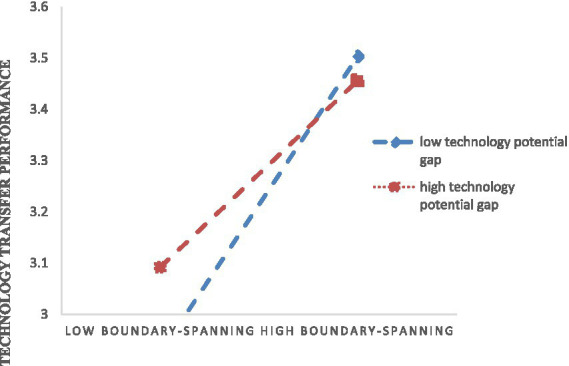
The figure of the moderator effect of technology potential gap.

As shown in Model 8, the interactive effect of technical potential gap and boundary-spanning behavior on technology transfer performance was significantly negative (*b* = −0.11, *p* < 0.01). We plotted the relationships between boundary-spanning behavior and technology transfer performance at high and low levels of potential technical difference (1 SD above and below the mean). In Figure 2, the simple slope tests indicate that boundary-spanning behavior on technology transfer performance is more vital for teams with lower levels of a potential technical difference than with high levels. This significant interaction effect supported Hypothesis 5.

**Figure 3:**
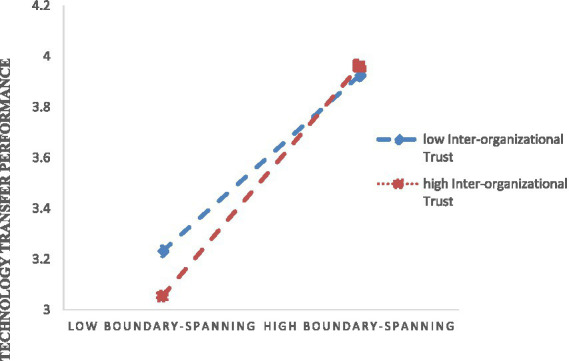
The figure of the moderator effect of inter-organizational trust.

In the meantime, we tested the interactive effect of trust and boundary-spanning behavior on technology transfer performance was significantly positive. We also plotted the relationships between boundary-spanning behavior and the technology transfer performance at high and low levels of trust. In Figure 3, the simple slope tests indicate that the positive effect of boundary-spanning behavior on technology transfer performance is more potent for teams with higher levels of trust rather than with lower levels. This significant interaction effect supported the hypothesis.

Based on the above analysis, we further hypothesized the existence of a mediating effect. First, it can be seen from Model 2 that policy perception ability has a significant positive impact on boundary-spanning behavior. Secondly, it can be seen from Model 5 that boundary-spanning behavior has a significant positive impact on the performance of enterprise technology transfer. Thirdly, technology potential gap and trust significantly moderate the relationship between boundary-spanning behavior and technology transfer performance. Based on the above conditions, policy perception ability has indirect effects on technology transfer performance through boundary-spanning behavior, and these indirect effects depend on the level of technology potential gap and inter-organizational trust. We conducted supplemental analyses to test the above hypothesis by calculating the 95% CI for the indirect effects conditioned at low (−1 SD) and high (+ 1 SD) potential technical differences as shown in Table 6. The results show that the indirect effect of policy perception on technology Transfer performance *via* boundary-spanning was not positive when the level of technology transfer potential was high [95%CI = (0.03, 0.11)], but it exists when the level of technology transfer potential was low [95% CI = (0.03, 0.29)]. Overall, it can be seen that boundary-spanning plays a mediating role when the potential technical difference is at the average or low level (excluding 0); in contrast, when the potential technical difference is high, the boundary-spanning does not play a mediating role (including 0). Based on the result, whether boundary-spanning plays a mediating role at the three levels is not consistent, so moderation has a mediating effect.

**Table 6:** Direct and mediating effects at different levels of technological potential gap and inter-organizational trust.

	Technological potential gap	Effect size	Boot SE	LLCI	ULCI
Direct effect	−1.45 (M−1SD)	0.32	0.06	0.19	0.45
	0.00 (M)	0.37	0.05	0.28	0.47
	1.45 (M + 1SD)	0.43	0.07	0.30	0.56
Mediating effect on boundary-spanning	−1.45 (M−1SD)	0.14	0.06	0.03	0.29
	0.00 (M)	0.08	0.03	0.03	0.16
	1.45 (M + 1SD)	0.03	0.03	0.00	0.11
	Trust	Effect size	Boot SE	LLCI	ULCI
Direct effect	−1.44 (M−1SD)	0.30	0.05	0.20	0.41
	0.00 (M)	0.47	0.05	0.36	0.57
	1.44 (M + 1SD)	0.63	0.08	0.48	0.78
Mediating effect on boundary-spanning	−1.44 (M−1SD)	0.09	0.04	0.03	0.18
	0.00 (M)	0.09	0.04	0.03	0.19
	1.44 (M + 1SD)	0.03	0.09	−0.16	0.21

Similarly, we conducted supplemental analyses by calculating the 95% CI for the indirect effects conditioned at low (−1 SD) and high (+ 1 SD) trust. The results show that the indirect effect of policy perception on technology transfer performance *via* boundary-spanning was positive when the level of trust was low [95%CI = (0.03, 0.18)] but not when the level of technology transfer potential gap was high [95% CI = (−0.16, 0.21)].

## Discussion

In the era of digital economy, technology transfer is a key factor affecting the technological innovation of enterprises, increasing the company’s general strength and competitive position (Ren and Huang, 2020). Our research constructs a “capability-behavior-performance” model, collects 402 questionnaires through field research in 42 technology-based enterprises in China, and uses MLR method (Multiple Linear Regression) to explore the complex mechanism of policy perception capability on technology transfer performance.

By combining social capital theory with previous policy perception researches, we found that policy perception has a positive effect on technology transfer performance. In fact, existing domestic and foreign studies on the driving factors of technology transfer performance are mostly from the perspective of the government, to explore the promotion of policies such as incentive systems, compensation systems and intellectual property protection on enterprise technology transfer performance (Zhang et al., 2013). Although we believe that incentive policies are important, the perception of policies within enterprises is equally important. The implementation of incentive policies is sometimes ineffective, or even contrary to the expected results, but this is not a problem of the policy itself, but the effect of the policy that is affected by the perceived differences of enterprises (Xin and Pearce, 1996; Li et al., 2016). Therefore, policy perception is the first and foremost premise for enterprises to be engage in the changing activities. Only in this way, enterprises perceive the intention of government policies, understand the connotation of policies, and actively participate in the application of policies and thus make profits, thereby improving the performance of enterprise technology transfer. Previous studies have also confirmed that the perception of government funding policies will motivate technology transfer academics to conduct cutting-edge research (Beaudry and Allaoui, 2012), and researchers who prefer practical applications to apply for more patents (Gulbrandsen and Smeby, 2005; Chen et al., 2021). These are all conducive to the improvement of technology transfer performance.

Besides, due to the different national conditions in economies, enterprises in different types of economies have diverse responses to the policies issued by the government. That is, most developing countries react differently to the publicity of government sector policies compared with developed countries (Peng, 2013). Since the financial crisis, developed countries have adjusted their industrial policies and increased policy support for technology R&D and industrialization in strategic areas such as intelligent manufacturing and artificial intelligence. Enterprises are more insensitive to those policies than developing countries due to its universality (Legrand, 2012; Jiang et al., 2022). In most developing countries, the situation is different. Some enterprises are passive recipients of policies, while others have established a good “government-enterprise relationship” with the government, acting as active policy advocates or even participating in the formulation of policies (Peng, 2013). Policy perception is sharply contrasting between those two kinds of enterprises in developing economy. Based on this, our research focuses on exploring different policy perception capabilities in the unique situation of developing countries. Moreover, our study contributes practical implications to exploring the impact of different policy perception capabilities of enterprises on technology transfer performance in developing countries.

Furthermore, in response to recent calls for a deeper understanding of policy perception capabilities (Yang Y. et al., 2022), this study further examines the mediating mechanism of boundary-spanning behavior between policy perception and technology transfer performance. Boundary-spanning behavior provides a favorable way for enterprises to achieve goals and acquire technology transfer resources. Enterprises with strong policy perception ability will stimulate the formation of target willingness. As a result, they should continue to cooperate with groups outside the company and exchange the latest policy information in the industry with partners promptly, discussing technology transfer bottlenecks and obstacles to improve technology transfer performance. The whole process further validates the technology acceptance model of Davis (1989).

Finally, we explore the effect of firm technology potential difference and trust level on the relationship between boundary-spanning behavior and technology transfer performance. Research has proved that the potential technical difference exerts a negative moderating effect between the two, while trust exerts a positive moderating effect. This requires enterprises to choose enterprises with little difference in technology potential difference from their own when engaging in transferring technology, which is conducive to the absorption of technology transfer knowledge. At the same time, enterprises can improve the trust between the two parties in the technology transfer process by improving their professionalism and establishing close contacts. The current study enriches the understanding and exploration of enterprise technology transfer paths.

### Contributions

This article makes several important contributions to the current literature. Firstly, our study advanced social capital theory, generating insight beyond what we know from existing technology transfer research. It has theoretical and practical significance for the complex mechanism that drives the improvement of enterprise technology transfer performance. Secondly, our study extends the impressive and growing body of research on technology transfer from a new perspective -- policy perception. The current research studying drive factors of enterprise technology transfer mainly focuses on the perspective of relationships, knowledge and structure, such as technological innovation networks (Liu and Yang, 2022), organizational proximity (Villani et al., 2017), knowledge collaboration (Villani et al., 2017), and endogenous evolutionary mechanisms (Broström, 2010; Slavtchev, 2013). Nevertheless，those researches have ignored the influence of policy perception as all businesses were running under the institutional environment, which is an essential research gap (Peng, 2013; Yang Z. et al., 2022). In this regard，starting from the antecedent variable of policy perception, this study clarifies the impact of policy perception usefulness and ease of use on enterprise technology transfer performance and its role path, expanding the application scenarios of technology transfer research and making up for the shortcomings of existing research. Besides, we explore how boundary-spanning, an important behavioral variable, exerts a mediating effect and explains behaviors of firms with higher policy perception take to influence technology transfer performance. Most previous studies have focused on the facilitation on organizational learning (Jian et al., 2012; Wu and Lin, 2022), knowledge sharing (Wang et al., 2021), technology transfer，ignoring the positive influence of boundary-spanning on technological resources acquisition, goal determination and risk reduction. Through social capital theory, we found that parties expand networks through boundary-spanning behaviors and acquire heterogeneous resources to break barriers for technology transfer, which is consistent with the research of some scholars (Wu et al., 2021; Janssen and Abbasiharofteh, 2022). Finally, our discussion on two moderating variables (i.e., technology potential difference and trust) clarifies the important characteristics that both sides of technology transfer should have, which are conducive to promoting the improvement of technology transfer performance by boundary-spanning.

### Limitations and future research directions

The study had several shortcomings, caused by various factors. First, the study was limited by the research method. This paper used questionnaires to measure technological potential gaps and technology transfer performance. Although previous scholars have revised the scale and claim it is universal and highly reliable, there may be flaws due to individual cognitive differences. Future research may want to use a combination of questionnaires and secondhand data, which would help prevent homologous variance. Second, the study was limited to Chinese enterprises operating in the context of China. Policies are an essential institutional variable that cannot be ignored when studying Chinese enterprises. It is appropriate to explore the impact of policy perception on technology transfer performance in the context of China, but due to the limited selection of the study sample, it may not be possible to generalize the study results to a wider population. Future scholars could explore the impact of the perception of different national policies on technology transfer performance. Furthermore, in this study we explored how the technological potential gap between two firms moderates policy perception and technology transfer performance. Future research could divide the samples into two groups (low potential and high potential) to explore their separate impacts on technology transfer performance—that is, whether high potential leads to better technology transfer performance or whether they have an inverted, U-shaped relationship.

### Conclusion

In the era of the digital economy, technology forms the core of an enterprise’s innovation and development. This trend makes it more difficult for enterprises to survive in the market and offers up more severe challenges than before. Technology transfer through technology sharing is conducive to technological innovation by enterprises. Specifically, it helps enterprises adopt new technology from other enterprises into their existing technical resources. This process is of important practical significance if enterprises want to gain a competitive advantage. Our study find the positive effect of policy perception capability (i.e., firms’ perception of the usefulness and ease of use) on technology transfer performance, which help enterprises have a detailed understanding between the ability of policy perception benefit from policies and generate a willingness to promote their participation in technology transfer.

Additionally, policy perception affects technology transfer performance through boundary-spanning behavior. Finally, technology potential gap plays a negative moderating effect on the relationship between boundary spanning and technology transfer performance, while inter-organizational trust has a positive moderating effect between boundary spanning and technology transfer performance. We hope that these studies can further promote the development of social capital theory and effectively alleviate the dilemma of technology transfer in developing countries in practical application.

## Data availability statement

The raw data supporting the conclusions of this article will be made available by the authors, without undue reservation.

## Author contributions

KZ drafted the manuscript. XW provided writing instruction. KZ, QW, and FZ collected, analyzed, and interpreted the data. QW instructed the project. All authors contributed to the article and approved the submitted version.

## Funding

This work was supported by The Natural Science Foundation of Shandong Province Project (ZR2018MG006).

## Conflict of interest

The authors declare that the research was conducted in the absence of any commercial or financial relationships that could be construed as a potential conflict of interest.

## Publisher’s note

All claims expressed in this article are solely those of the authors and do not necessarily represent those of their affiliated organizations, or those of the publisher, the editors and the reviewers. Any product that may be evaluated in this article, or claim that may be made by its manufacturer, is not guaranteed or endorsed by the publisher.
